# Hemp Inflorescence as a Sustainable Biostimulant Tool to Boost Growth and Antioxidant Capacity in Oilseed Pumpkin

**DOI:** 10.3390/plants14223473

**Published:** 2025-11-14

**Authors:** Ivana Varga, Manda Antunović, Monika Tkalec Kojić, Antonela Markulj Kulundžić, Dario Iljkić, Renata Baličević, Marija Ravlić

**Affiliations:** 1 Department of Plant Production and Biotechnology, Faculty of Agrobiotechnical Sciences Osijek, Josip Juraj Strossmayer University of Osijek, Vladimira Preloga 1, 31000 Osijek, Croatia; manda.antunovic@fazos.hr (M.A.); monikat@fazos.hr (M.T.K.); dario.iljkic@fazos.hr (D.I.); 2 Department of Industrial Plants Breeding and Genetics, Agricultural Institute Osijek, 31000 Osijek, Croatia; antonela.markulj@poljinos.hr; 3 Department of Phytomedicine, Faculty of Agrobiotechnical Sciences Osijek, Josip Juraj Strossmayer University of Osijek, Vladimira Preloga 1, 31000 Osijek, Croatia; renata.balicevic@fazos.hr (R.B.); marija.ravlic@fazos.hr (M.R.)

**Keywords:** organic agriculture, phytoextracts, *Cannabis sativa* L. inflorescences extract, root, stem, growth parameters, *Cucurbita pepo* var. hull-less, antioxidant activity

## Abstract

The study investigates whether water extracts from industrial hemp inflorescences influence the germination and early growth of hull-less oilseed pumpkin (*Cucurbita pepo* L.), with the hypothesis that industrial hemp extracts may act as a biostimulant, enhancing growth, biomass, and bioactive compound accumulation in pumpkin seedlings. Fully developed and healthy inflorescences of industrial hemp were harvested, dried, ground into powder, filtered, and diluted to concentrations of control (water), 1.0%, 2.5%, and 5.0% for the seed germination bioassay. Morphological, growth parameters, and bioactive compounds of the hull-less oilseed pumpkin sprouts were determined. Total germination rate was not affected with industrial hemp inflorescent water extracts, while sprout vigor index and biomass increased at 2.5 and 5.0% of the extract applied. The average root length of hull-less oilseed pumpkin sprouts was 14.19 cm, the stem length was 5.45 cm, and the fresh mass of the sprouts was 14.10 g per plant. Water extracts of 2.5 and 5.0% significantly (*p* ≤ 0.001) increased stem length by more than double, and the sprouts’ fresh mass by about 35% compared to the control. The average *Chl a* (chlorophyll), *Chl b*, *Chl a* + *b,* and *Car* (carotenoids) content was on average 0.161, 0.115, 0.268, and 0.136 mg g^−1^ FW, respectively, and were significantly affected compared to the control. The highest total phenol (TPC) and flavonoid content (TFC) were determined for hull-less oilseed pumpkin sprouts at 1.0% of water extract (100.21 µg QC/1 g tissue and 0.02 µg GA/1 g tissue, respectively). Low absolute values are consistent with the early seedling stage, where secondary metabolism is underdeveloped. The antioxidant activity was determined with the FRAP (Ferric Reducing Antioxidant Power) method and a significant influence (*p* ≤ 0.05) of industrial hemp inflorescence water extracts on antioxidant activity of pumpkin sprouts was observed, which significantly (*p* ≤ 0.05) increased on all treatments compared to the control, by 36% on average, with no significant differences among different concentrations of water extracts. Overall, industrial hemp inflorescence water extracts have a positive influence on the observed parameters, supporting the potential use of industrial hemp inflorescence water extracts as a biostimulant for hull-less oilseed pumpkin.

## 1. Introduction

Many plants have been part of folk medicine for centuries, especially due to their medicinal purposes. There is an increasing use of plant extracts in agriculture, mainly in organic farming and sustainable alternative systems to traditional agriculture, which use many medicinal, aromatic, and herbal plants for multiple roles, e.g., in the regulation of plant diseases, insects, herbicides, allelopathic use, biostimulants, growth retardant, etc. [1,2,3,4,5,6,7,8]. Godlewska et al. [9] state that different plant parts can have varying influences, and seeds, fruits, flowers, stems, leaves, and roots can be used to produce plant-based extracts. Nowadays, chemical fertilizers play a major role in increasing crop yields and food production. However, their excessive and long-term use has led to several issues such as loss of fertility, structure, and microbial life, as well as runoff of nitrogen and phosphorus into the soil and water [10,11,12]. Biostimulants have become increasingly important in agricultural production, especially because they can reduce the amount of chemical fertilizers needed, thereby lowering input costs [13,14,15]. Biostimulants are substances of various organic or inorganic origins and chemical compositions that exert a biostimulator effect on plants. Application of biostimulants such as amino acids, humic extracts, and seaweed extracts, helps mitigate the impact of stressful conditions and enhances plant resistance to both abiotic and biotic stress factors [16,17,18,19,20]. Special benefits can be found in the use of natural sources, as the plant extracts are eco-friendly and most often cost-effective. The use of natural sources can bring special benefits, improve crop resilience and help plants cope with drought, salinity, and extreme temperatures [21,22,23].

Industrial hemp (*Cannabis sativa* L.) is increasingly attracting attention due to its multi-purpose applications and environmental sustainability [24,25,26,27]. As a fast-growing crop with low pesticide and water requirements, hemp is used to produce fibers, seeds, oil, bioplastics, construction materials, and bioactive compounds such as cannabidiol (CBD). It is mostly permitted to cultivate industrial hemp with a total delta-9-tetrahydrocannabinol (THC) content of 0.2% to 1.0% [28,29,30]. Industrial hemp has significant development potential within the framework of sustainable agriculture and bioeconomy.

Hull-less oilseed pumpkin (*Cucurbita pepo* subsp. *pepo*) seeds are usually used as a raw material for cold-pressed oil, because seeds contain about 40–50% of oil [31,32,33]. Except for oil, there is a great advantage to human consumption, since the seeds do not require de-hulling, they are used as a snack and in the baking industries [34]. Many farmers grow it in Europe, especially in Austria, Poland, the Czech Republic, Hungary, Slovenia, Serbia, Romania, and Slovakia [35,36,37,38,39,40,41]. The seeds are dark green and have a high content of free fatty acids and oil and are often used in salads. The seeds are rich in vitamins, healthy fatty acids, phytosterols, magnesium, zinc, selenium, and carotenoids [42,43,44,45,46]. Seeds also contain cucurbitacins—compounds with antitumor and antimicrobial properties [46,47].

Both seed germination and early seedling development are influenced by a range of factors. The speed of the sprouts’ growth and development depends on several factors: quality, temperature of the substrate and air, water supply, and lighting conditions [48,49,50,51,52]. First, seeds need to absorb water and swell, and under the influence of moisture, the seed coat softens, and the conditions necessary for germination and emergence are activated. The success of germination in vegetables, flowers, and other plant species is most influenced by the ability to absorb water, i.e., swelling and heat [53]. There are several environmental factors such as heat, cold, disease attack, drought, acidity, salinity, etc., all of which can significantly affect the speed and uniformity of germination and early seedling growth [54]. Seedling or sprout vigor reflects the health status and growth rate, both of which are critical for successful establishment and subsequent crop performance. During this early stage, seedlings rely heavily on the nutrient reserves stored within the seeds, as they are not yet fully photosynthetically active and have not developed a sufficiently extensive root system to absorb nutrients and water from the soil.

Wazeer et al. [55] stated that protein-based biostimulants are typically applied via foliar sprays or soil irrigation; however, their use in seed priming has recently emerged as a promising and sustainable approach to enhance seed quality and germination. Use of different types of extracts as biostimulants in organic agriculture is increasing in order to achieve sustainable and environmentally friendly food production [56,57]. Plant-based biostimulants enhance plant metabolism, nutrient uptake, and resistance to abiotic stress without introducing synthetic chemicals into the agroecosystem. Skowronek et al. [58] reported that industrial hemp extracts significantly increased the activity of antioxidant enzymes, extending the life of bees (*Apis mellifera*). Jolayemi et al. [59] highlight the growing scientific importance of protein-based biostimulants, which activate natural physiological processes to enhance plant growth. These biostimulants are typically derived from plant or animal sources, often as by-products or waste materials from agro-industrial processes, an approach that is particularly significant in the context of the circular bioeconomy and sustainability [55,56,57,58,59,60].

Plant extracts can have a two-way effect; they can inhibit plant growth and development, but they can also promote plant growth or growth at certain phases of development. Although certain effects of plant species on each other are known, there are still many unknowns when it comes to the interaction of different plant species and their influence on plant growth and development. The effects of plant extracts varied depending on whether fresh or dry biomass was used and whether the extracts exhibited stimulatory or inhibitory activity. Since higher concentrations (generally ≥5%) often showed inhibitory effects, concentration proved to be a key factor influencing the response. According to the literature, plant extracts are usually used in small portions generally ≥5%, and not higher than 10% [5,8,8,23,61,62,63].

Industrial hemp is still, in many cases, an underutilized crop with great potential for organic farming. Its inflorescences are rich in bioactive compounds such as terpenes, phenolics, flavonoids, and phytocannabinoids, many of which are known to have antioxidant, antimicrobial, allelopathic, or biostimulant effects on plants [64,65,66,67]. Moreover, hemp is a sustainable crop that grows quickly, requires minimal inputs, and improves soil structure, making it well-suited for use in ecological and regenerative agriculture. Its low THC content of industrial hemp genotypes also makes it safe and legally acceptable for research and field application.

Hull-less oilseed pumpkin is a crop of increasing economic and nutritional value, particularly in organic systems where its seeds are used for oil production and as a functional food. In professional and organic farming, pumpkins are often grown from seedlings, rather than direct seeding, to achieve better early establishment, uniform growth, and earlier harvest [68]. However, young seedlings are more vulnerable to environmental stress (e.g., drought, salinity, pathogens), and the use of natural biostimulants during this sensitive stage can significantly support plant development and resilience.

Based on the above, this study aimed to determine the effect of the industrial hemp inflorescence water extract of different concentrations on germination and early growth of the hull-less oilseed pumpkin during the sprouts phase.

## 2. Results

### 2.1. One-Way ANOVA

According to the results of the one-way ANOVA of the present study, the industrial hemp inflorescence water extracts significantly influenced (*p* ≤ 0.05) oilseed pumpkin root growth (Table 1). The stem and total sprout length, as well as the fresh mass per plant of oilseed pumpkin sprouts, were very significantly influenced (*p* ≤ 0.001) by the industrial hemp inflorescence water extracts. There was a significant influence of industrial hemp inflorescence water extracts on *Car* content.

The total phenol content of the hull-less oilseed pumpkin sprouts was significantly (*p* ≤ 0.05) influenced by the industrial hemp water extracts (Table 1), whereas total flavonoid content in the hull-less oilseed pumpkin sprouts was very significantly (*p* ≤ 0.01) affected by the industrial hemp inflorescence water extracts. The antioxidant activity measured by the FRAP method differed significantly (*p* ≤ 0.05) in the hull-less oilseed pumpkin sprouts under the influence of different water extracts.

For sprout growth parameters of the hull-less oilseed pumpkin, there was a significant influence (*p* ≤ 0.05) of industrial hemp inflorescence water extracts determined for the vigor index (VI), and a significant (*p* ≤ 0.05) influence for root-to-shoot ratio (RSR) and sprout biomass index (SBI) (Table 1).

### 2.2. Morphological and Growth Parameters of the Hull-Less Oilseed Pumpkin Sprouts

**Figure 1:**
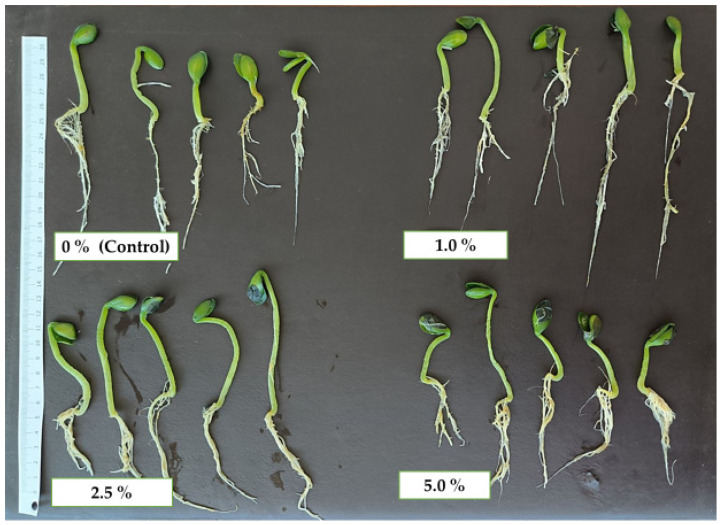
Hull-less oilseed pumpkin sprouts regarding different concentrations of industrial hemp inflorescence water extracts.

**Figure 2:**
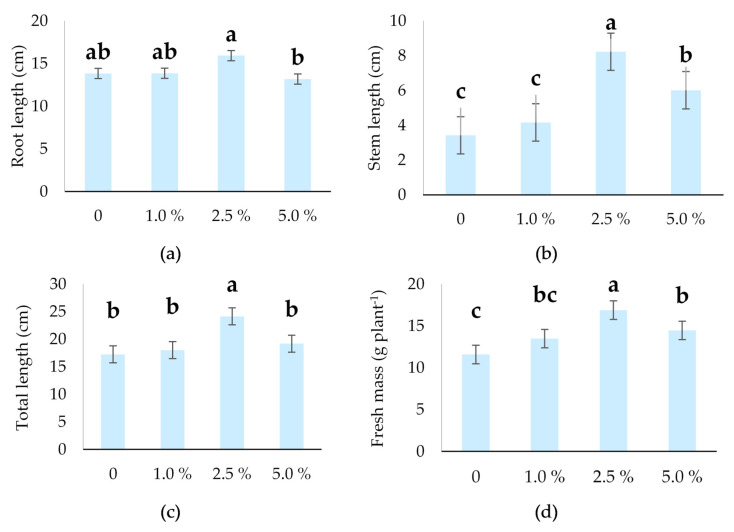
Morphological parameters of oilseed pumpkin sprouts regarding different concentrations of industrial hemp inflorescence water extracts: (**a**) stem length (cm), (**b**) root length (cm), (**c**) total length (cm), and (**d**) fresh mass (g plant ^−1^). Different letters above the bars indicate statistically significant differences at *p* ≤ 0.05 (one-way ANOVA). Vertical error bars represent the standard error (SE) of the mean at 5%.

The results concerning the morphological parameters of oilseed pumpkin sprouts are presented in Figure 1 and Figure 2. The highest average root length (Figure 1 and Figure 2a) was observed at the 2.5% treatment (15.92 cm), which was significantly greater than the 5.0% treatment (13.17 cm). However, it was not significantly different from the 0% (13.83 cm) and 1.0% (13.85 cm) treatments. The 5.0% treatment showed the lowest root length and was the only one statistically different from the highest value, suggesting that higher concentrations may negatively affect root growth.

The longest stem was measured at the 2.5% treatment (8.22 cm), which was significantly higher than all other treatments (Figure 2b). The 5.0% treatment (6.01 cm) was significantly lower than 2.5%, but higher than both the control, 0% (3.42 cm), and 1.0% industrial hemp inflorescence water extracts (4.16 cm), which did not differ significantly from each other. This suggests that moderate treatment levels may enhance the measured outcome, while higher or lower concentrations reduce the effect.

The total length of oilseed pumpkin sprouts differs significantly (*p* ≤ 0.001) from the industrial hemp inflorescence water extracts (Table 1). The 2.5% treatment resulted in the longest sprouts (24.14 cm), which was significantly greater than all other treatments (Figure 2c). The industrial hemp inflorescence water extracts 0%, 1.0%, and 5.0% showed no significant differences among each other.

The fresh mass of the oilseed pumpkin sprouts was also significantly different among the different concentrations of industrial hemp inflorescence water extracts (Table 1). The longest sprouts at the 2.5% treatment also showed the highest fresh mass (16.88 g/plant), which was significantly greater than all other treatments (Figure 2d). The 0% treatment had the lowest value of the fresh mass per plant (11.58), while the sprouts’ fresh mass at 1.0% and 5.0% were intermediate and not significantly different from each other.

### 2.3. Total Germination Rate and Growth Parameters of the Hull-Less Oilseed Pumpkin Sprouts

**Figure 3:**
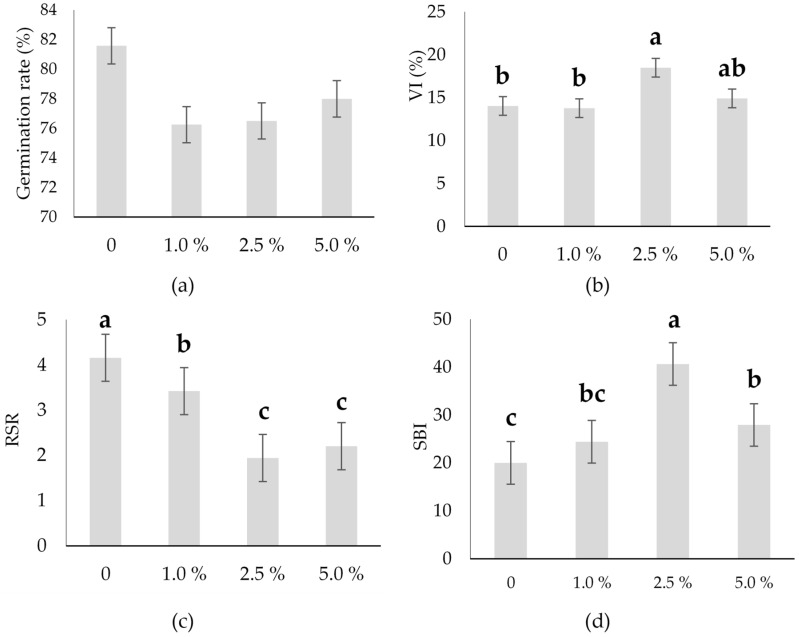
Germination rate and growth parameters of hull-less oilseed pumpkin sprouts regarding different concentrations of industrial hemp inflorescence water extracts: (**a**) germination rate (%), (**b**) vigor index (VI) (%), (**c**) root-to-shoot ratio, and (**d**) sprout biomass index. Different letters above the bars indicate statistically significant differences at *p* ≤ 0.05 (one-way ANOVA). Vertical error bars represent the standard error (SE) of the mean at 5%.

There was no significant difference determined for total germination rate (Table 1), even though industrial hemp inflorescence water extracts had a mild inhibitory effect on seed germination across all tested concentrations (1–5%) in comparison to the control (Figure 3a).

The greatest reduction was recorded at 1%, but differences between the treated groups were relatively small. The highest germination rate, 82%, was recorded at the control treatment (0%), while all treatments with the extract showed a reduction in germination. At 1.0%, the germination rate dropped to 76%, which was the lowest value observed.

In the present study, at the 2.5% industrial hemp inflorescence water extract, the VI was the highest (~19%), which was significantly greater than the control and 1.0% treatment (Figure 3b). The RSR of the hull-less oilseed pumpkin sprouts was 2.93 (Table 1) and was very significantly (*p* ≤ 0.001) affected by the industrial hemp inflorescence water extract. The highest RSR was found at the control (2.16), and the lowest at 2.5% industrial hemp inflorescence water extract (1.94) (Figure 3c). The SBI was also significantly (*p* ≤ 0.001) affected by the industrial hemp inflorescence water extract (Table 1). The lowest SBI was found at the control (19.99), and the highest at the 2.5% industrial hemp inflorescence water extract (40.64) (Figure 3d).

### 2.4. The Pigment Status of the Hull-Less Oilseed Pumpkin Sprouts

Industrial hemp inflorescence water extracts had a very significant (*p* ≤ 0.001) influence on the average *Chl a* content of oilseed pumpkin sprouts (Table 1).

**Figure 4:**
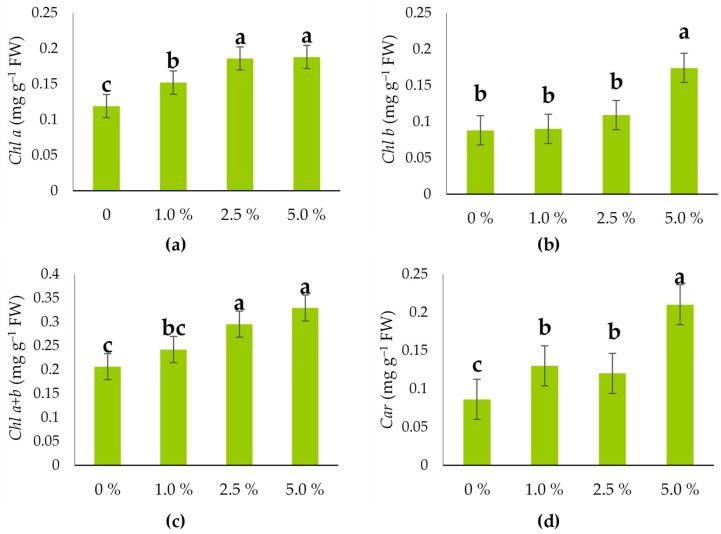
Pigment content of hull-less oilseed pumpkin sprouts (FW) at different levels of industrial hemp inflorescence water extracts: (**a**) *Chl a* (mg g^−1^ FW), (**b**) *Chl b* (mg g^−1^ FW), (**c**) *Chl a* + *b* (mg g^−1^ FW), and (**d**) *Car* (mg g^−1^ FW). Different letters above the bars indicate statistically significant differences at *p* ≤ 0.05 (one-way ANOVA). Vertical error bars represent the standard error (SE) of the mean at 5%.

The average *Chl a* content of oilseed pumpkin sprouts was 0.161 mg g ^−1^ FW. Industrial hemp inflorescence water extracts had a positive influence on increasing *Chl a* of the oilseed pumpkin sprouts (Figure 4a). Control (0%) had the lowest *Chl a* content (~0.125 mg g^−1^ FW), 1.0% extract had a moderate increase in *Chl a* (~0.145 mg g^−1^ FW), while the highest content was determined at 2.5% and 5% (0.187 mg g^−1^ FW on average), but with no significant difference between the latter two concentrations.

A very significant (*p* ≤ 0.001) influence of industrial hemp inflorescence water extracts was found for *Chl b* content (Table 1). The average *Chl b* content was 0.115 mg/g FW (Figure 4b). Industrial hemp inflorescence water extracts positively influenced the *Chl b* content in oilseed pumpkin sprouts, which was the highest at 5% (0.174 mg/g FW).

The content of *Chl a* + *b* has also been significantly (*p* ≤ 0.05) affected by the industrial hemp water extracts (Table 1). The average content was 0.268 mg/g FW (Figure 4c). The lowest value was recorded in the control (0%), at 0.206 mg g^−1^ FW, and increased up to 0.329 mg g^−1^ FW at 5% of industrial hemp inflorescence water extracts.

The average *Car* content was 0.136 mg/g FW (Table 1). The highest *Car* content was observed at 5.0% (0.210 mg/g FW), suggesting a strong stimulatory effect at higher concentrations (Figure 4d).

### 2.5. Total Phenols, Flavonoids, and Antioxidant Activity of the Hull-Less Oilseed Pumpkin Sprouts

The TPC was significantly (*p* ≤ 0.05) influenced by the industrial hemp water extracts (Table 1). An average of 0.017 µg GA/1 g tissue content in oilseed pumpkin sprouts was found for the TPC (Table 1). Also, the TFC in the oilseed pumpkin sprouts was significantly influenced (*p* ≤ 0.01) by the industrial hemp inflorescences’ water extracts (Table 1).

**Figure 5:**
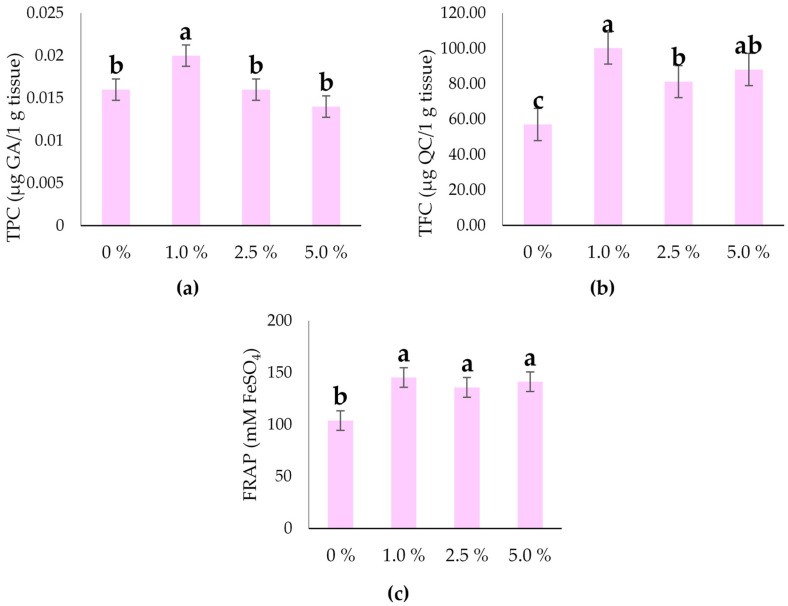
Total phenols (TPC), flavonoids (TFC), and antioxidant activity of hull-less oilseed pumpkin sprouts (FW) at different levels of industrial hemp inflorescence water extracts: (**a**) TPC (µg GA/1 g tissue), (**b**) TFC (µg QC/1 g tissue), and (**c**) antioxidant activity—FRAP (mM FeSO_4_). Different letters above the bars indicate statistically significant differences at *p* ≤ 0.05 (one-way ANOVA). Vertical error bars represent the standard error (SE) of the mean at 5%.

The treatment with 1.0% resulted in the highest TPC (Figure 5a), approximately 0.020 µg GA/g tissue, which is significantly higher than all other groups. In contrast, the control group (0%) and the treatments with 2.5% and 5.0% showed lower and statistically similar values, indicating no significant difference among them.

The 1.0% treatment significantly increased QC concentration compared to all other treatments (Figure 5b). At the 2.5% and 5.0% industrial hemp inflorescence water extract treatments were not significantly different from each other for TFC, but the 2.5% was significantly lower than the 1.0% (Figure 5a).

Figure 5b shows the TFC under different treatments. The treatment with 1.0% resulted in the highest TFC level, approximately 100 µg QC/g tissue, which is statistically different from the other groups. In contrast, the control group (0%) exhibited the lowest TFC (57.03 µg QC/g tissue). The 2.5% treatment showed a significantly higher TFC compared to the control but lower than the 1.0% treatment, suggesting that increasing the concentration beyond 1.0% does not further enhance TFC levels and may even result in a slight decrease.

Antioxidant activity was significantly affected by the industrial hemp inflorescence water extracts (Table 1), being the lowest in oilseed pumpkin sprouts in the control (103.886 mM FeSO_4_), while all treatments with industrial hemp inflorescence water extracts increased the antioxidant activity of oilseed pumpkin sprouts (Figure 5c).

### 2.6. Correlation Analysis

**Figure 6:**
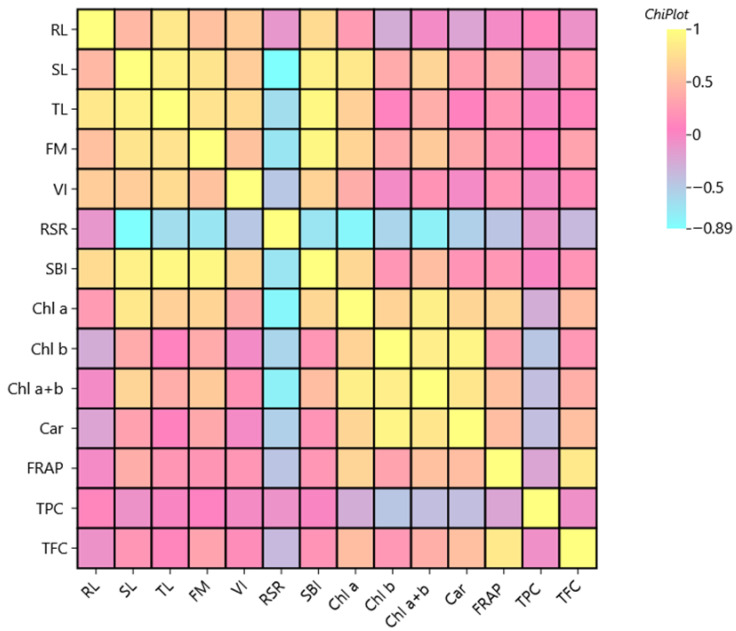
The heatmap of Pearson correlation coefficient for total gemination, morphological parameters, bioactive compounds, and antioxidant activity of the hull-less oilseed pumpkin sprouts (TG—total germination rate, RL—root length, SL—stem length, TL—total sprout length, FM—fresh mass, VI—vigor index, RSR—root to stem ratio, SBI—sprouts biomass index, FRAP—antioxidant activity FRAP (mM FeSO_4_), TPC—total phenol content, TFC—total flavonoid content). Each cell shows the Pearson correlation coefficient (r) between two variables, which ranges from: +1.0 (perfect positive correlation), 0 (no correlation), and –1.0 (perfect negative correlation) (N = 16).

To determine the relationship among all measured parameters, the Pearson correlation coefficient was determined (Table S1). To visually identify which parameters responded similarly or oppositely under industrial hemp inflorescence water extract treatment, a heat map was created (Figure 6). The heat map illustrates the interconnection between morphological growth, pigment synthesis, and antioxidant activity in seedlings exposed to industrial hemp extract.

Total germination (TG) showed no significant correlations with most other parameters in the present study (Figure 6), except for a weak positive correlation with fresh mass (FM) (r = 0.576; *p* ≤ 0.05) (Table S1). Shoot length (SL) showed strong positive correlations with TL (r = 0.887 **), FM (r = 0.797 **), SBI (r = 0.888 **), and VI (r = 0.626 **). This indicates that shoot elongation is a central trait reflecting seedling performance under extract influence.

The shoot length (SL) also showed a strong negative correlation with root to shoot ratio (RSR) (r = –0.890, *p* ≤ 0.01), indicating that as shoot growth increases, the proportion of biomass allocated to roots decreases (Table S1). The root length (RL) was positively correlated with hull-less oilseed pumpkin sprouts’ total length (TL) (r = 0.823; *p* ≤ 0.001), fresh mass (r = 0.541; *p* ≤ 0.05), and the vigor index (VI) (r = 0.632; *p* ≤ 0.001), confirming its importance in overall seedling growth.

*Chl a* was strongly correlated with *Chl b* (r = 0.668, *p* ≤ 0.01), *Chl a* + *b* (r = 0.877, *p* ≤ 0.01), and carotenoids (r = 0.689, *p* ≤ 0.01), as expected due to their shared role in photosynthesis (Table S1). *Chl b* and *Car* had a particularly strong correlation (r = 0.916, *p* ≤ 0.001), suggesting coordinated pigment synthesis.

The antioxidant capacity of the hull-less oilseed pumpkin sprouts determined with the FRAP method was significantly positively correlated with total TFC (r = 0.840, *p* ≤ 0.001), and moderately with TPC, confirming the major role of flavonoids in antioxidant activity (Table S1, Figure 6).

## 3. Discussion

### 3.1. Morphological Parameters and Growth Analysis of the Hull-Less Oilseed Pumpkin Sprouts

There is still a lack of studies involving industrial hemp as a plant-based biostimulant in agricultural production. Du Jardin [68] states that biostimulants, including plant-based biostimulants, are increasingly used worldwide. With the present study, the influence on hull-less pumpkin sprouts is evident and statistically confirmed. Even though the total germination rate was not significantly influenced by the industrial hemp water extracts’ inflorescence, other growth parameters were significantly affected by their application. Similarly, total germination did not show significant correlation with the majority of the parameters (Figure 6, Table S1). This suggests that although germination rate itself was not directly associated with shoot/root length or biochemical traits, seedlings that did germinate tended to produce more biomass under certain conditions. Thus, the results of the total germination rate of the present study suggest that certain bioactive compounds present in the industrial hemp inflorescence water extract may moderately interfere with physiological processes involved in seed germination. The VI responded non-linearly to increasing concentrations of hemp extract. Interestingly, the 2.5% extract concentration significantly enhanced seedling vigor, suggesting a possible stimulatory effect at moderate doses. In contrast, both lower (1.0%) and higher (5.0%) concentrations showed reduced vigor, possibly indicating inhibitory or phytotoxic effects at suboptimal and elevated extract levels. Islam et al. [69] found that industrial hemp was used as seed priming media, and among the 14 hemp varieties, Han FNQ (China) showed the highest seed germination and seedling vigor, while low-concentration ClO_2_ pre-treatment (500 mg·L^−1^) improved early seedling growth despite a general decline in germination across all pre-treatments.

According to Andrić [70], seed aging is one of the main causes of reduced vigor and poor field emergence, which becomes especially evident under adverse environmental conditions such as drought, low temperatures, or soil compaction, ultimately leading to decreased crop yield and quality. For the cultivar of oilseed pumpkin with the hull, in media with various pH values (2.5–8.5), Varga et al. [71] found an average germination rate of 92%, with differences among pH values, reflecting the plant’s stress response to acidic and alkaline media. The highest germination rate was observed at pH 8.5 (94%), followed by a total of 93% at pH 2.5, 4.5, and 7.5. Lynch et al. [72] state that root architecture reflects the most stressful conditions in the soil systems. Additionally, authors stated that root-to-shoot ratio usually varies widely among plant species, and it is highly modified by external factors. In the present study, the significantly lower RSR of hull-less oilseed pumpkin sprouts was at 2.5% of industrial hemp inflorescence water extract, which may also align with the earlier observation of increased VI at that same concentration, potentially reflecting balanced or prioritized shoot elongation in favorable conditions. A decrease in the RSR at the concentration of 2.5% and 5.0% (Figure 2c) suggests that these treatments led to relatively greater shoot growth or inhibition of root development, thereby lowering the root-to-shoot proportion. This shift could indicate a physiological stress response at specific extract concentrations.

### 3.2. Leaf Pigments, Phenols, Flavonoid Content, and Antioxidant Capacity of the Hull-Less Oilseed Pumpkin Sprouts

The average *Chl a* content in the hull-less oilseed pumpkin sprouts of the present study was 0.161 mg g^−1^ FW. The application of industrial hemp inflorescence water extracts significantly enhanced *Chl a* level, with maximum values observed at concentrations of 2.5% and 5.0%. These findings indicate a positive and concentration-dependent stimulatory effect, particularly up to 2.5%. The results suggest that industrial hemp inflorescence water extracts significantly enhance *Chl a* level in oilseed pumpkin sprouts. The most significant increase in *Chl b* was observed at the 5.0% concentration, suggesting a concentration-dependent stimulation of *Chl a* biosynthesis. There are several studies that also confirm the increase in leaf pigment status with the application of biostimulants. Francesca et al. [73] stated that application of biostimulant CycoFlow (Agriges, Benevento, Italy) on different tomato genotypes significantly influences the changes of Chl *a*, *Chl b*, *Car*, and ascorbic acid in a way that *Chl a* content is consistently higher than *Chl b* across both control and with biostimulant application, while *Car* levels remained relatively stable across treatments. In potato (*Solanum tuberosum*) leaves, Mystkowska [74] found that the application of biostimulants increased the SPAD (Soil–Plant Analysis Development) leaf greenness index and plant height in all three potato cultivars compared to the control. Di Mola et al. [75] found that in baby rocket leaf (*Diplotaxis erucoides* L., cv. ‘Reset’) treated with foliar applications of biostimulators, one a legume-derived protein hydrolysate and the other a tropical plant extract, there was a significant increase of 112.0% and 79.2%, respectively, in total ascorbic acid, with no significant difference on lipophilic antioxidant activity. The authors stated that *Chl a* and *Chl b* content increased with both biostimulants, as compared to the control. Carotenoids, a class of terpenoids functioning as antioxidants and photosynthetic pigments, are positively affected by various environmental and biochemical factors [76,77]. In our study, the highest carotenoid content (0.210 mg g^−1^ FW) was recorded in hull-less oilseed pumpkin sprouts at the 5.0% of the industrial hemp water extract concentration, indicating a strong stimulatory effect of higher biostimulant levels on carotenoid accumulation in the sprouts.

According to the correlation analysis of the present study, the heatmap illustrates the strength and direction of relationships (correlations) between various morphological, physiological, and biochemical parameters of seedlings treated with industrial hemp extract (Figure 6). Moderate to strong positive correlations were found between growth indices (e.g., FM, TL, VI) and pigment or antioxidant traits (e.g., *Chl a*, Car, FRAP), possibly reflecting stress adaptation or industrial hemp inflorescence water extract influence. No strong correlation was observed between FRAP and total *Chl* content, indicating that pigment synthesis and antioxidant mechanisms may be independently regulated under the tested conditions. In our study, 1.0% concentration was optimal for enhancing TPC (Figure 4a), while higher concentrations (2.5% and 5.0%) did not provide additional benefit and resulted in a return to baseline levels observed in the control. Moreover, in the present study, the 1.0% treatment appears to be the most effective concentration for enhancing TFC (Figure 5b), suggesting that higher concentrations of industrial hemp inflorescence (2.5% and 5.0%) do not provide additional benefits and may indicate a saturation point or possible inhibitory effects at elevated levels.

The TPC in plants plays a key role in plant defense, growth regulation, and stress response. Kalinová et al. [78] stated that the levels of flavonoids such as rutin, orientin, and vitexin in the cookies and pasta made from wheat and common buckwheat sprouts or microgreens are influenced by multiple factors, including the food matrix, heating method, temperature, processing duration, interactions with other compounds, and the chemical properties of the flavonoids themselves. Total Flavonoid Content (TFC) represents the combined amount of all different flavonoid compounds found in the plant tissue.

In the present study, the antioxidant activity of hull-less oilseed pumpkin (Figure 4c) was increased in all treatments, which was significantly higher (*p* ≤ 0.05) compared to the control. These findings highlight the positive impact of biostimulants on enhancing the antioxidant potential in plants. Relatively low concentrations of TPC and TFC observed in this study are due to the early developmental stage of the seedlings. At this stage, secondary metabolism is still underdeveloped, as the plants prioritize primary growth processes. In contrast, older seedlings, transplants, or mature plants typically accumulate higher levels of these compounds, which explains the differences reported in other studies. Posmyk and Szafrańska [79] state that biostimulants can enhance plant tolerance to adverse environmental conditions, including chilling, drought, salinity, chemical pollutants, and heavy metal stress. For tomato plants (*Solanum lycopersicum* L., cv.‘Coronel’) Cozzolino et al. [80] found that the application of plant-based biostimulants (an extract of brown seaweed *Ecklonia maxima* (Osbeck), legume derived protein hydrolysate, and a tropical plant extract), significantly affected lipophilic antioxidant activity, hydrophilic antioxidant activity, and ascorbic acid content, compared with an untreated control, while no statistically significant differences were observed in total phenol content. To evaluate the biostimulant effects of biological preparation based on rosemary (*Salvia rosmarinus* L.) and eucalyptus (*E. globulus* L.) essential oils, Chrysargyris et al. [81] used it for tomato seedlings (cv. Brillande Grade A). The authors stated that the ascorbic acid content of the fruits was markedly enhanced by application of biostimulant (147.02 mg 100 g^−1^ FW), more than doubling the control value, and that phenol content was highest in foliar spray with the *Razormin* commercial product treated (2.59 μmol GAE g^−1^ FW). On the other hand, plant extracts can have an inhibitory influence on plant germination and bioactive compounds. Plant extracts, particularly at higher concentrations, may release allelopathic compounds that interfere with seed germination, and could potentially be used for natural weed control or bioherbicide development, though with caution regarding crop safety. Teacă et al. [82] tested different concentrations (0–0.2 g/L) of ethanol extracts from *Melissa officinalis* (lemon balm, V1 and V2) and *Lavandula angustifolia* (lavender, V3) on the germination percentage of various crop seeds, including wheat, rye, triticale, soybean, and bean. Authors stated that all three plant extracts exhibit allelochemical effects, reducing seed germination in a dose-dependent manner, in a way that the wheat was consistently the most resilient species across all treatments, while legumes (soybean and bean) and triticale appeared most susceptible to the inhibitory effects. Authors stated that lemon balm extract has the strongest inhibitory effect, likely due to higher concentrations or different types of active compounds (e.g., phenolics, terpenoids).

In the present study, a concentration of 2.5% industrial hemp inflorescence water extract improved growth parameters such as root, stem, and total length, as well as fresh mass. However, for leaf pigments, the 5.0% concentration showed the best results for *Chl a*, *Chl b*, *Chl* (*a* + *b*), and *Car*. The FRAP antioxidant activity increased in all treatments compared to the control, but the highest TPC and total flavonoid content TFC were observed at the 1.0% concentration. In the context of sustainable agriculture and the growing demand for environmentally friendly production systems, there is increasing interest in identifying natural biostimulants that can promote plant growth, enhance stress tolerance, and improve crop quality—all without the use of synthetic agrochemicals.

For other plant species and water extracts, Mutlu-Durak et al. [83] stated that water extracts of weeping willow leaves and bark, rich in bioactive compounds such as salicylates and phenolics, were shown to act as effective plant-based biostimulants for maize, enhancing shoot fresh weight, root area, and leaf protein content. Yadav et al. [84] examine twenty-five herbal extracts for their biostimulant activity on wheat through seed priming, assessing morphological parameters such as germination, seedling growth, and biomass, as well as biochemical traits including phenolics, flavonoids, enzyme activity, and antioxidant capacity. Extracts from *Phyllanthus emblica*, *Plumbago zeylanica*, *Catharanthus roseus*, and *Baccopa monnieri* were particularly effective, with principal component analysis grouping the samples based on their growth-promoting activity. Although it remains difficult to predict how findings of our study would translate into field conditions for pumpkin cultivation, it opens up new opportunities for further research, especially due to many growers producing pumpkins as seedlings in controlled environments, where the biostimulant effect could be more effective.

## 4. Materials and Methods

### 4.1. Preparation of Water Extracts

The full, expanded, and healthy inflorescences of industrial hemp (cv. Finola) were collected at the full flowering stage on 5 July 2024, from the experimental field of the Faculty of Agrobiotechnical Sciences Osijek, located in Tenja, Croatia. The inflorescence was used for water extract preparation in the present study. The harvested inflorescences were oven-dried (Kambič, SP-440,Semič, Slovenia) for 48 h. Once completely dried, the biomass was chopped and ground using an electric mill (Retsch, Haan, Germany) into a fine powder that passed through a 1 mm sieve. The preparation of *C. sativa* water extracts was based on the method described by Norsworthy [85], with slight modifications. Specifically, 50 g of the dried industrial hemp inflorescence powder were soaked in 1000 mL of distilled water (1:20 *w*/*v*) at room temperature (21 ± 2 °C) for 24 h. The mixture was initially filtered through a fine cloth to remove coarse particles, followed by two rounds of filtration through filter paper (Munktell, Bärenstein, Germany), to obtain a stock solution with a 5% concentration, reflecting the ratio of plant material to solvent used in preparation. This stock extract was then diluted with distilled water to achieve final working concentrations of 0% (control), 1.0%, 2.5%, and 5.0% for the seed germination bioassay in Petri dishes. All extracts were freshly prepared one day before the start of the experiment and stored at 4 °C to prevent microbial growth and preserve the integrity of bioactive compounds.

### 4.2. Seed Germination Bioassay in Petri Dishes

The experiment was conducted under controlled conditions at the Faculty of Agrobiotechnical Sciences Osijek (Croatia). The experiment was carried out using a completely randomized design with four replications per treatment. Each replicate consisted of a separate sterilized Petri dish (90 mm diameter) lined with a double layer of filter paper (Munktell 80 g/qm, Germany). For the experiment, the seeds of hull-less oilseed pumpkin (*Cucurbita pepo* L.), variety “Štrigovec” (Croatian plant genetic resources database Accession number IND00071 [86]), collected from the project National program for the conservation and sustainable use of plant genetic sources for food and agriculture in the Republic of Croatia, were used. For each replicate, 10 seeds of hull-less oilseed pumpkin were evenly distributed on the filter paper.

The filter paper was moistened with 10 mL of industrial hemp (*Cannabis sativa* L.) inflorescence water extract, applied in three concentrations: 1.0%, 2.5%, and 5.0%. The 0% treatment (distilled water) served as the solvent control. Seeds were incubated at 21 ± 2 °C under a 12 h light/12 h dark photoperiod.

### 4.3. Total Germination and Morphological Parameters of Hull-Less Oilseed Pumpkin Sprouts

On the final day (14th day) of the experiment, the total germination rate percentage for each treatment was calculated using the equation: G (%) = (Number of germinated seeds/Total number of seeds) × 100. The root and shoot lengths of seedlings were measured manually using a ruler, and the fresh mass of seedlings was recorded using a precision balance. To assess early seedling performance, the Vigor Index (VI) was calculated according to the following equation: VI = (Average seedling length (cm) × Germination rate percentage)/100. The Root-to-Shoot Ratio (RSR) was determined according to the equation: Mean root length (cm)/Mean stem length (cm). Sprouts Biomass Index (SBI) was calculated using the equation: SBI = Fresh weight (g per plant) × Total seedling length (cm).

Fresh hull-less oilseed sprouts were put in the laboratory freezer at −80 °C for further analysis of the bioactive compounds.

### 4.4. Analyses of the Pigment Content of the Hull-Less Oilseed Pumpkin Sprouts

To determine photosynthetic pigment content, 0.05 g of the powdered hull-less oilseed pumpkin sprouts extract was accurately weighed into a 15 mL test tube, followed by the addition of 10 mL of acetone. The mixture was homogenized using a vortex mixer and subsequently centrifuged at 4000 rpm for 10 min at 4 °C. From the resulting supernatant, 2 mL was transferred into a cuvette, and absorbance was measured at A662 nm, A644 nm, and A440 nm using a spectrophotometer (Shimadzu, UV-1800, Kyoto, Japan).

The concentrations of chlorophyll a (*Chl a*), chlorophyll b (*Chl b*), total chlorophyll (*Chl a* + *b*), and carotenoids (*Car*) were calculated using the Holm–Wettstein [87,88] equations, expressed in mg dm^−3^:

*Chlorophyll a* (*Chl a*) = 9.784 × A662 − 0.990 × A644

*Chlorophyll b* (*Chl b*) = 21.426 × A644 − 4.650 × A662

*Total chlorophyll* (*a* + *b*) (*Chl a* + *b*) = 5.134 × A662 + 20.436 × A644

*Carotenoids* (*Car*) = 4.695 × A440 − 0.268 × (*Chl a* + *b*)

To express the results per gram of fresh weight (mg/g FW), the concentration in the extract was multiplied by the extract volume (dm^3^) and divided by the fresh weight of the plant material (g) used for extraction. This provides the pigment content relative to the fresh mass of the sample.

### 4.5. Analyses of the Total Phenolic and Flavonoid Content of the Industrial Hemp Water Extract and Hull-Less Oilseed Pumpkin Sprouts

The TPC in the industrial hemp water extract and hull-less oilseed pumpkin sprouts was determined using the Folin–Ciocalteu method as described by Singleton and Rossi [89], with minor modifications in three technical replications. The reaction mixture consisted of the sample extract, distilled water, Folin–Ciocalteu reagent, and sodium carbonate (Na_2_CO_3_). Following incubation at 37 °C for 60 min, absorbance was measured at A765 nm using a spectrophotometer (Shimadzu, UV-1800, Japan) in three technical repetitions. A standard calibration curve (y = 2.939x + 0.0677, R^2^ = 0.9953) was generated using increasing concentrations of gallic acid, and results were expressed as gallic acid equivalents (mg GAE/g FW).

The total flavonoid content was determined in the ethanol extract after the addition of aluminum chloride (AlCl_3_) and 96% ethanol in three technical replications. The reaction mixture was homogenized and incubated at room temperature for 60 min, after which absorbance was measured at 415 nm. A calibration curve (y = 0.0076x + 0.0304, R^2^ = 0.9988) was prepared using standard solutions of quercetin, and results were expressed as quercetin equivalents (mg QC/g FW).

For the industrial hemp inflorescence water extracts, the total phenolic content was 0.0128 µg GA/1 g tissue, and total flavonoid content was 30.52 µg QC/g tissue. The results were recalculated from mg GAE/g FW and mg QC/g FW to µg GA/g and µg QC/g for consistency in the tables.

### 4.6. Antioxidant Activity of the Hull-Less Oilseed Pumpkin Sprouts

The antioxidant capacity of the hemp extract was assessed using the Ferric Reducing Antioxidant Power (FRAP) assay, following the method described by Keutgen and Pawelzik [90], with slight modifications. The FRAP reagent was added to the ethanol extract of the sample, and the reaction mixture was incubated at 37 °C for 4 min. Subsequently, the absorbance was measured at A593 nm using a spectrophotometer (Shimadzu, UV-1800, Japan).

A standard calibration curve (y = 1.2461x + 0.0505, R^2^ = 0.9969) was prepared using increasing concentrations of FeSO_4_, and the antioxidant capacity was expressed as mM FeSO_4_ per gram of fresh weight (mM FeSO_4_ g^−1^ FW).

### 4.7. Biometric Approach

All obtained data were transferred into MS Excel (MS Office 2011), and afterward, the SAS Enterprise Guide, 7.1, was used for biometric analysis. The one-way ANOVA was used to determine the differences in total germination, morphological parameters, and bioactive compounds. The normality of data distribution was tested using the Shapiro–Wilk test, while Bartlett’s test was applied to verify the homogeneity of variances. The influence of industrial hemp inflorescence water extracts on hull-less oilseed pumpkin early growth was expressed as the significance: ns—not significant, * *p* ≤ 0.05, ** *p* ≤ 0.01, *** *p* ≤ 0.001. In the case of the significant F value, the post hoc Student t-test (Least Significant Difference, LSD test) was used to determine the differences between the means. There were three degrees of freedom (DF) for all parameters, and eight error degrees of freedom (DF).

The Pearson correlation coefficient was used to determine correlations. To visualize the relationships among all parameters in the present study, a correlation heat map was drawn using *ChiPlot* [91] (https://www.chiplot.online/) (accessed on 30 July 2025).

## 5. Conclusions

The use of biostimulants derived from plant extracts offers a sustainable and environmentally friendly approach to crop management, particularly within the context of organic agriculture, which is experiencing significant global growth. Hull-less oilseed pumpkin is a crop often cultivated under organic practices, and the use of plant-based biostimulants can play an important role in the growing process. Even though antioxidant activity was primarily driven by flavonoid content, not directly by seedling morphology or pigment levels, the water extract of industrial hemp inflorescence significantly influenced all bioactive compound content in the hull-less oilseed pumpkin sprouts. The promising results also highlight the need for follow-up greenhouse and field experiments to evaluate practical applicability.

Although different concentrations had different strengths, the 2.5% industrial hemp water extract can be considered the most effective overall, as it significantly enhanced plant growth and biomass, which are key indicators of vitality. Meanwhile, the 5.0% concentration was most beneficial for enhancing photosynthetic pigments, and 1.0% was best for increasing certain antioxidant compounds.

Industrial hemp shows considerable potential in this regard, and future research should further explore its applications, including for other crop species. Further research is recommended to include other arable crops, as well as various parts of the industrial hemp plant. Such studies could contribute to a more sustainable and efficient use of agricultural resources and potentially reveal new functionalities and value-added uses for hemp and other crop by-products.

## Figures and Tables

**Table 1:** The one-way ANOVA and mean values of the total germination rate, morphological parameters, sprout growth parameters, bioactive compounds, and antioxidant activity of the hull-less oilseed pumpkin sprouts.

Parameter	Unit	Mean Square	F Value	*p* > F	LSD_0.05_	Mean
Root length		5.682	3.10	*	2.09	14.19
Stem length	cm	18.376	23.27	***	0.91	5.45
Total length		38.41	16.34	***	2.36	19.64
Fresh mass	g per plant	0.195	9.09	***	0.23	14.10
Germination rate	%	24.759	0.21	ns	-	78.08
VI		18.994	2.99	*	3.88	15.30
RSR		4.347	12.79	***	0.898	2.93
SBI		315.67	18.99	***	6.282	28.21
*Chl a*	mg/g FW	0.003	39.06	***	0.017	0.161
*Chl b*		0.004	20.77	***	0.029	0.115
*Chl a* + *b*		0.009	8.36	*	0.062	0.268
*Car*		0.008	28.38	***	0.032	0.136
FRAP	mM FeSO_4_	1067.38	5.26	*	24.833	131.57
TPC	µg GA/1 g tissue	0.001	1.39	*	0.006	0.017
TFC	µg QC/1 g tissue	992.25	13.24	**	16.297	81.65

VI—Vigor index, SBI—seedlings biomass index; *Chl*—chlorophyll, FRAP—Ferric Reducing Antioxidant Power, TPC—total phenols content, TFC—total flavonoids content. Significance: ns—not significant * *p* ≤ 0.05, ** *p* ≤ 0.01, *** *p* ≤ 0.001.

## Data Availability

Data are contained within the article.

## Supplementary Materials

The following supporting information can be downloaded at: https://www.mdpi.com/article/10.3390/plants14223473/s1, Table S1. The correlation analysis of the hull-less oilseed pumpkin sprouts’ morphological and growth parameters, bioactive compounds, and antioxidant activ-ity (N = 16).

## Abbreviations

TG	total germination rate
RL	root length
SL	stem length
TL	total sprout length
FM	fresh mass
FW	Fresh weight
*Chl a*	Chlorophyll *a*
*Chl b*	Chlorophyll *b*
*Car*	Carotenoids
VI	Vigor index
RSR	Root to shoot ratio
SBI	Sprouts biomass index
SE	Standard Error
FRAP	ferric reducing antioxidant power
TPC	total phenol content
TFC	total flavonoid content
