# Supplementary material for: Hemp Inflorescence as a Sustainable Biostimulant Tool to Boost Growth and Antioxidant Capacity in Oilseed Pumpkin
Source: Plants (Basel). 2025 Nov 14;14(22):3473. doi: 10.3390/plants14223473 (PMC12655846; doi:10.3390/plants14223473)
Supplement: Supplementary file 1 [file plants-14-03473-s001.zip › plants-3948532-supplementary.pdf]

Table S1. The correlation analysis of the hull-less oilseed pumpkin sprouts' morphological and growth parameters, bioactive compounds, and antioxidant activity (N=16)

	TG	RL	SL	TL	FM	VI	RSR	SBI	Chl a	Chl b	Chl a+b	Car	FRAP	TPC	TFC
TG	1	-0.071	-0.131	-0.121	-0.144	0.576	0.048	-0.143	-0.245	-0.142	-0.204	-0.141	0.014	-0.021	0.081
		ns	ns	ns	ns	*	ns	ns	ns	ns	ns	ns	ns	ns	ns
RL		1	0.468	0.823	0.541	0.632	-0.119	0.729	0.251	-0.272	-0.036	-0.218	-0.029	0.110	-0.078
			ns	***	*	***	ns	***	ns	ns	ns	ns	ns	ns	ns
SL			1	0.887	0.797	0.626	-0.890	0.888	0.825	0.368	0.680	0.301	0.391	-0.077	0.222
				***	***	***	***	***	***	ns	**	ns	ns	ns	ns
TL				1	0.795	0.733	-0.635	0.952	0.645	0.080	0.400	0.067	0.225	0.012	0.094
					***	**	**	***	**	ns	ns	ns	ns	ns	ns
FM					1	0.543	-0.701	0.940	0.680	0.368	0.608	0.352	0.215	0.055	0.316
						*	**	***	**	ns	**	ns	ns	ns	ns
VI						1	-0.475	0.673	0.389	-0.033	0.195	-0.028	0.218	-0.014	0.154
							ns	ns	ns	ns	ns	ns	ns	ns	ns
RSR							1	-0.691	-0.829	-0.577	-0.782	-0.539	-0.453	-0.083	-0.372
								**	**	*	**	ns	ns	ns	ns
SBI								1	0.696	0.215	0.517	0.199	0.223	0.018	0.207
									*	ns	ns	ns	ns	ns	ns
Chl a									1	0.668	0.877	0.689	0.683	-0.278	0.511
										*	***	*	*	ns	ns
Chl b										1	0.870	0.916	0.315	-0.468	0.235
											***	***	ns	ns	ns
Chl a+b											1	0.813	0.537	-0.410	0.406
												***	ns	ns	ns
Car												1	0.511	-0.407	0.524
													ns	ns	ns

FRAP	1	-0.217<br>ns	0.840<br>***
TPC		1	-0.054<br>ns
TFC			1

ns – not significant	*** $p \leq 0.001$	** $p \leq 0.01$	* $p \leq 0.05$

TG – total germination rate, RL – root length, SL – stem length, TL – total sprout length, FM – fresh mass, VI – vigor index, RSR – root to stem ratio, SBI – sprouts biomass index, FRAP – antioxidant activity FRAP (mM $FeSO_4$), TPC – total phenol content, TFC – total flavonoid content
